# Boromycin Kills Mycobacterial Persisters without Detectable Resistance

**DOI:** 10.3389/fmicb.2016.00199

**Published:** 2016-02-22

**Authors:** Wilfried Moreira, Dinah B. Aziz, Thomas Dick

**Affiliations:** Antibacterial Drug Discovery Laboratory, Department of Microbiology and Immunology, Yong Loo Lin School of Medicine, National University of SingaporeSingapore, Singapore

**Keywords:** boromycin, membrane-targeting antibiotic, tuberculosis, resistance, persistence

## Abstract

Boromycin is a boron-containing polyether macrolide antibiotic isolated from *Streptomyces antibioticus.* It was shown to be active against Gram positive bacteria and to act as an ionophore for potassium ions. The antibiotic is ineffective against Gram negative bacteria where the outer membrane appears to block access of the molecule to the cytoplasmic membrane. Here we asked whether boromycin is active against *Mycobacterium tuberculosis* which, similar to Gram negative bacteria, possesses an outer membrane. The results show that boromycin is a potent inhibitor of mycobacterial growth (MIC_50_ = 80 nM) with strong bactericidal activity against growing and non-growing drug tolerant persister bacilli. Exposure to boromycin resulted in a rapid loss of membrane potential, reduction of the intracellular ATP level and leakage of cytoplasmic protein. Consistent with boromycin acting as a potassium ionophore, addition of KCl to the medium blocked its antimycobacterial activity. In contrast to the potent antimycobacterial activities of the polyether macrolide, its cytotoxicity and haemolytic activity were low (CC_50_ = 30 μM, HC_50_ = 40 μM) with a selectivity index of more than 300. Spontaneous resistant mutants could not be isolated suggesting a mutation frequency of less than 10^-9^/CFU. Taken together, the results suggests that targeting mycobacterial transmembrane ion gradients may be an attractive chemotherapeutic intervention level to kill otherwise drug tolerant persister bacilli, and to slow down the development of genetic antibiotic resistance.

## Introduction

*Mycobacterium tuberculosis*, the causative agent of Tuberculosis (TB), remains the biggest bacterial killer with 1.3 million deaths per year (Zumla et al., 2014). Emergence of genetic drug resistance and persistence of infection despite extensive chemotherapy represent two major issues in current TB treatment regimens (Barry et al., 2009; Gengenbacher and Kaufmann, 2012). TB drugs, like most antibiotics, target specific macromolecules. Antibiotic resistance arises mainly from mutations in target genes (Cohen et al., 2014). Genetic resistance occurs readily in patients due to non-compliance to lengthy (6–24 months) multi-drug regimens and spatio-temporal pockets of monotherapy (Prideaux et al., 2015). Persistence of infection is thought to be due to various types of metabolically quiescent non-growing drug tolerant bacteria. Several culture models have been developed to study persister bacilli *in vitro*. A first type of mycobacterial persister may pre-exist in growing cultures or is induced upon treatment of growing cultures with drugs. Isoniazid for instance shows a bi-phasic kill pattern (Ahmad et al., 2009; de Steenwinkel et al., 2010). An initial rapid kill is followed by a phase of reduced killing leaving intact a small subpopulation of drug tolerant persisters (Keren et al., 2011). A second type of persisters can be grown *in vitro* by exposing a replicating culture to adverse culture conditions. Growth is terminated and the non-replicating organisms display drug tolerance (Dartois et al., 2009; Sarathy et al., 2013). The Wayne model of persistence (Wayne and Hayes, 1996) for instance exposes the obligate aerobe to hypoxia, a micro-environmental condition demonstrated for TB lesions *in vivo* (Lenaerts et al., 2015). Upon oxygen depletion the whole growing culture shifts down to a state of non-replicating persistence. A current working model suggests that killing both persister types may be required to achieve treatment shortening (Dick, 2001; Barry et al., 2009). Ideally new TB drugs should kill persister bacilli and show a low propensity for the development of genetic drug resistance.

**FIGURE 1:**
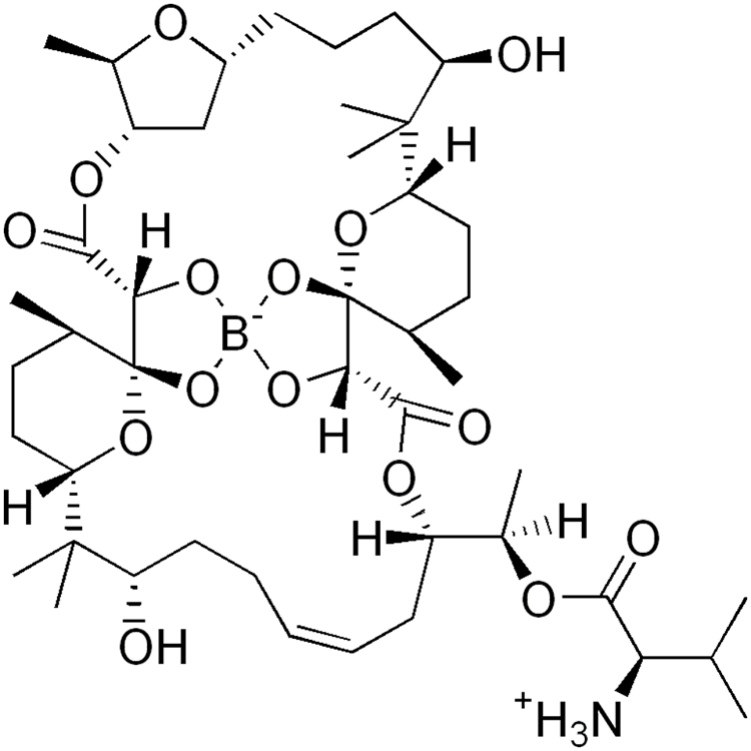
**Structure of boromycin (Hutter et al., 1967)**.

Bacterial membrane integrity and its associated homeostatic functions, such as maintenance of ion gradients, is essential regardless of the metabolic status of the cell (Hurdle et al., 2011). The large number of antimicrobial peptides made by the host validates the membrane as an antibacterial target site and successful clinical use of a few membrane-targeting antibiotics, supports this notion (Hurdle et al., 2011). Furthermore, resistance development to membrane-targeting antibiotics is expected to be slow as the target is the product of complex biosynthetic pathways, as opposed to a single macromolecule. This notion is supported by the slow emergence of resistance against membrane targeting antibiotics used in the clinic (Hurdle et al., 2011). Current TB drugs do not target the membrane. Here, we used boromycin, an old polyether macrolide antibiotic isolated from *Streptomyces antibioticus* to ask whether targeting the mycobacterial membrane may be a chemotherapeutic intervention level that would achieve both killing of non-growing persister bacilli and slowing down the development of genetic drug resistance. Boromycin was the first natural product and the first antibiotic found to contain the trace element boron (Hutter et al., 1967). Its structure was elucidated by degradation studies and X-ray analysis (Dunitz et al., 1971) and is shown in **Figure 1**. Boromycin’s *in vitro* bactericidal activity was first reported in 1967 against Gram positive bacteria. The antibiotic was shown to act as ionophore causing cellular potassium ion loss leading to growth arrest and cell death. The compound is ineffective against Gram negative bacteria where the outer membrane appears to block access of the compound to the cytoplasmic membrane (Hutter et al., 1967; Pache and Zahner, 1969).

## Materials and Methods

### Strains, Culture Conditions, and Chemicals

*Mycobacterium tuberculosis* H37Rv (ATCC 27294) and *M. bovis* BCG (ATCC 35734) liquid cultures were grown in Middlebrook 7H9 broth (BD Difco) supplemented with 0.5% albumin, 0.2% glucose, 0.085% sodium chloride, 0.5% glycerol, 0.05% Tween 80. Solid medium cultures were grown on Middlebrook 7H10 agar (BD Difco) supplemented with 0.5% albumin, 0.2% glucose, 0.085% sodium chloride, 0.5% glycerol, 0.0003% catalase, and 0.006% oleic acid as described previously (Lakshminarayana et al., 2015). *Staphylococcus aureus* (ATCC 29213), *S. epidermidis* (ATCC 14990), *Enterococcus faecalis* (ATCC 29212), *Escherichia coli* (ATCC 25922), *Acinetobacter baumannii* (ATCC19606), and *Klebsiella pneumonia (*ATCC BAA-1705) liquid cultures were grown in LB media (BD Difco). To generate hypoxic non-growing mycobacterial cultures, bacteria were grown in sealed glass tubes with stirring as described previously (Lim et al., 1999). HepG2 (ATCC HB.8065) and Vero (ATCC CCL-81) cells were cultured at 37°C with 5% CO_2_ atmosphere in DMEM media (Gibco) complemented with 10% FBS heat-inactivated (Gibco), penicillin (100 U/mL, Gibco) and streptomycin (100 μg/mL, Gibco). Red blood cells were obtained from Interstate Blood Bank, Inc. Laboratory, USA. Boromycin was purchased from AdipoGen. Carbonyl cyanide m-chlorophenyl hydrazone (CCCP), 3-(4,5-dimethylthiazol-2-yl)-2,5-diphenyltetrazolium bromide (MTT), Triton X-100, and isoniazid were purchased from Sigma–Aldrich.

### Growth Inhibition and Bactericidal Assays

Mininum Inhibitory Concentrations (MICs) were determined in broth as described previously by the broth dilution method (Murugasu-Oei and Dick, 2000; Gopal and Dick, 2015; Lakshminarayana et al., 2015) using turbidity (optical density) as a proxy for growth (Murugasu-Oei and Dick, 2000). We report MIC_50_ and MIC_90_, the concentrations that inhibit 50 and 90% of growth, respectively, as compared to the untreated control. Bactericidal activities, i.e., ‘Minimal bactericidal concentration’ (MBC_99_) for growing bacteria, and ‘Wayne cidal concentration’ (WCC_99_) for hypoxic non-growing bacilli were determined as described previously (Murugasu-Oei and Dick, 2000; Lakshminarayana et al., 2015). Controls of Wayne’s model of hypoxia included treatment with 5 μM of isoniazid or moxifloxacin resulting in no any bactericidal activity and 10 fold killing, respectively. Exponentially growing cultures or hypoxic non-growing cultures were exposed to various drug concentrations for 5 days and then plated for CFU (colony forming unit) determination.

### Spontaneous Resistant Mutant Selection

*Mycobacterium bovis* BCG was grown to mid log phase (O.D. 600 = 0.4–0.6) and10^8^ CFU were plated per 7H10 standard agar plate containing one, two or four times the MIC_90_ concentration of boromycin. In order to reach 10^9^ CFU and not overload the plates, we plated 10 times 10^8^ CFU on 10 different plates. The plates were incubated for a total of 8 weeks (Gopal and Dick, 2015).

### Time-Kill, Cell-Content Release, Membrane Potential, and ATP Level Determinations

Time-kill curves were determined as follows: 10^7^ CFU inoculum of *M. bovis* BCG were treated with MIC_90_ of boromycin or Isoniazid and samples were taken at various time points for CFU determination on agar plates. To assess the effect of boromycin on the release of cell content, we transformed *M. bovis* BCG with plasmid pGMEH-P38-mRFP (Kim et al., 2011) (pGMEH-P38-mRFP was a gift from Dirk Schnappinger, Addgene plasmid # 27058) encoding mCherry Red Fluorescent Protein (mRFP) under the control of a strong promoter. We subjected this strain for 2 days to MIC_90_ of INH, boromycin or left it untreated. Supernatant was collected by two serial centrifugations. Fluorescence was measured using Tecan Infinite M200Pro plate reader (λ587/630 nm). Similarly, the effect of boromycin on the membrane potential was measured using the Baclight Bacterial Membrane Potential Kit (Life Technologies) according to the supplier’s recommendations. ATP level was determined using the BactiterGlo kit (Promega) according to manufacturer’s recommendations.

### Cytotoxicity Assays

The MTS (Promega) reduction assay was used to assess HepG2 (ATCC HB-8065) or Vero (ATCC CCL-8) cell viability after exposure to boromycin according to manufacturer’s guidelines. Absorbance was read with a Tecan M200Pro plate reader at 570 nm. Optical density (O.D.) was plotted as a function of drug concentration and cytocidal concentrations were determined. CCCP was used as a cytotoxic positive control. Hemolytic activity of boromycin was evaluated by exposing 10^6^ red blood cells to two fold serial dilutions of compounds in phosphate-buffered saline for 24 h. Red blood cells were subsequently centrifuged at 1000 g for 10 min and the supernatant was collected and homogenized by vortexing. Absorbance was read at 540 nm. Triton X-100 treated control was used to define 100% of hemoglobin release.

## Results

### Boromycin Kills Growing and Non-growing Persister Mycobacteria, Shows a High Selectivity Index, and No Detectable Resistance

We first determined whether boromycin shows anti-mycobacterial activity or whether the outer membrane of mycobacteria would, similar to the outer membrane in Gram negative bacteria, cause intrinsic resistance to the antibiotic. Virulent *M. tuberculosis* H37Rv and its closely related, BSL2 compatible, cousin *M. bovis* BCG were used as testing strains. The broth dilution method was employed to determine MIC_50_ and MIC_90_ (minimum inhibitory concentrations that inhibit 50 and 90% of growth) by turbidity measurements and MBC_99_ (minimum bactericidal concentration that causes a 100 fold kill) was obtained by recovering CFU on agar (Murugasu-Oei and Dick, 2000). We also determined WCC_99_ (minimum bactericidal concentration that causes a 100-fold kill in the Wayne model), i.e., the concentration that kills hypoxic non-replicating persister bacilli (Lakshminarayana et al., 2015). **Table 1** shows that boromycin displayed an attractive growth inhibition potency with MIC_50_ values of around 80 nM and bactericidal activity against both replicating and non-replicating tubercle bacilli with identical MIC_90_, MBC_99_, and WCC_99_ values of 200 nM. These results show that boromycin possesses very potent anti-mycobacterial activity despite the presence of an outer membrane in this group of bacteria and that its cidal activity is independent of the replication status of the bacilli, which means that the antibiotic kills not only aerated growing cultures but also hypoxic non-growing cultures (Supplementary Figure S1). **Table 1** also shows that boromycin displayed potent growth inhibition and bactericidal activity against Gram positive pathogens including *S. aureus, S. epidermidis*, and *E. faecalis* but was inactive against Gram negative bacteria including *E. coli, A. baumannii*, and *K. pneumoniae* confirming previous reports (Hutter et al., 1967).

**Table 1:** Antibacterial and cytotoxic activities of Boromycin.

	Antibacterial activity (μM)			
	MIC_50_	MIC_90_	MBC_99_	WCC_99_
*M. tuberculosis* H37Rv	0.08	0.2	0.2	nd
*M. bovis* BCG	0.05	0.2	0.2	0.2
*S. aureus*	0.1	0.3	0.6	na
*S. epidermidis*	0.1	0.3	0.3	na
*E. faecalis*	0.15	0.2	0.4	na
*E. coli*	>100	>100	na	na
*A. baumannii*	>100	>100	na	na
*K. pneumoniae*	>100	>100	na	na

	**Cytotoxicity (μM)**			
	**CC_50_**		**CC_90_**	

HepG2	35		>50	
Vero	25		>50	

	**Haemolytic activity (μM)**			
	**HC_50_**		**HC_90_**	

Human red blood cells	40		>50	

*MIC_50_/MIC_90_, minimum inhibitory concentrations that inhibit 50 and 90% growth of replicating culture, respectively. MBC_99_, minimum bactericidal concentration that kills 99% of replicating culture. WCC_99_, Wayne bactericidal concentration that kills 99% of hypoxic non-replicating culture. CC_50_/CC_90_, cytocidal concentration that kills 50 and 90% of HepG2 and Vero cells, respectively. HC_50_/HC_90_, haemolytic concentration that induces lysis of 50 and 90% of red blood cells, respectively. n.d., not determined; n.a., not applicable.*

To determine boromycin’s selectivity index, we measured cytotoxicity against mammalian HepG2 and Vero cells using a tetrazolium reduction viability assay (Riss et al., 2004), and its haemolytic activity against human red blood cells using an hemoglobin-release assay (Dausset and Contu, 1967). Boromycin’s cytocidal (CC_50_) and haemolytic (HC_50_) concentrations (concentrations that kills 50% of HepG2 or Vero cells and induces the lysis of 50% of red blood cells), were shown to be 35, 25, and 40 μM, respectively, (**Table 1**). These results suggest that boromycin has a high selectivity for the mycobacterial membrane with a selectivity index (CC_50_/MIC_50_) of more than 300.

**FIGURE 2:**
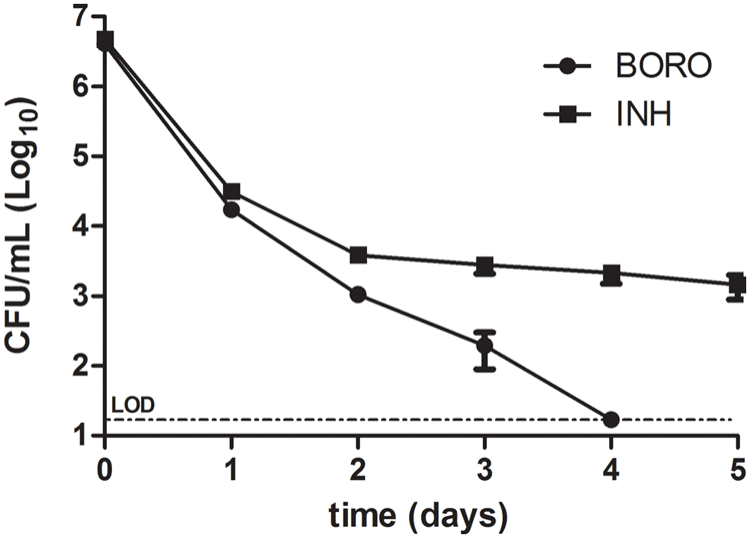
**Time-kill curve of boromycin compared to Isoniazid.** Exponentially growing cultures of *Mycobacterium bovis* BCG were treated with boromycin (BORO, 0.2 μM = 1x MIC_90_) or Isoniazid (INH, 0.5 μM = 1x MIC_90_) and CFU were enumerated at the time points indicated. Experiments were conducted three times. Shown are the averages with standard deviations. LOD, limit of detection (30 CFU/ml).

To determine whether boromycin kills growing cultures completely or whether the antibiotic shows a – for many TB drugs typical – biphasic kill curve, time-kill experiments were conducted in which we quantified the reduction of CFU as a function of exposure time. **Figure 2** shows the typical biphasic kill curve of isoniazid. After an initial reduction of CFU the curve levels off and leaves intact a population of viable non-growing persister bacteria. In contrast boromycin showed a monophasic kill curve resulting in an apparent sterilization of the culture.

To determine spontaneous resistance mutation frequencies for boromycin we plated twice 10^9^ CFU logarithmic phase *M. bovis* BCG cultures on agar plates containing one, two or four times the MIC_90_ of the antibiotic. No boromycin resistant colonies could be identified even after extended incubation of the selection plates for 8 weeks. This result shows that the spontaneous resistance mutation frequency of the tubercle bacillus against boromycin is smaller than 10^-9^/CFU. In contrast Isoniazid selection delivered resistant strains at a frequency of 10^-6^/CFU.

### Boromycin Acts as Potassium Ionophore and Causes Collapse of the Membrane Potential

**FIGURE 3:**
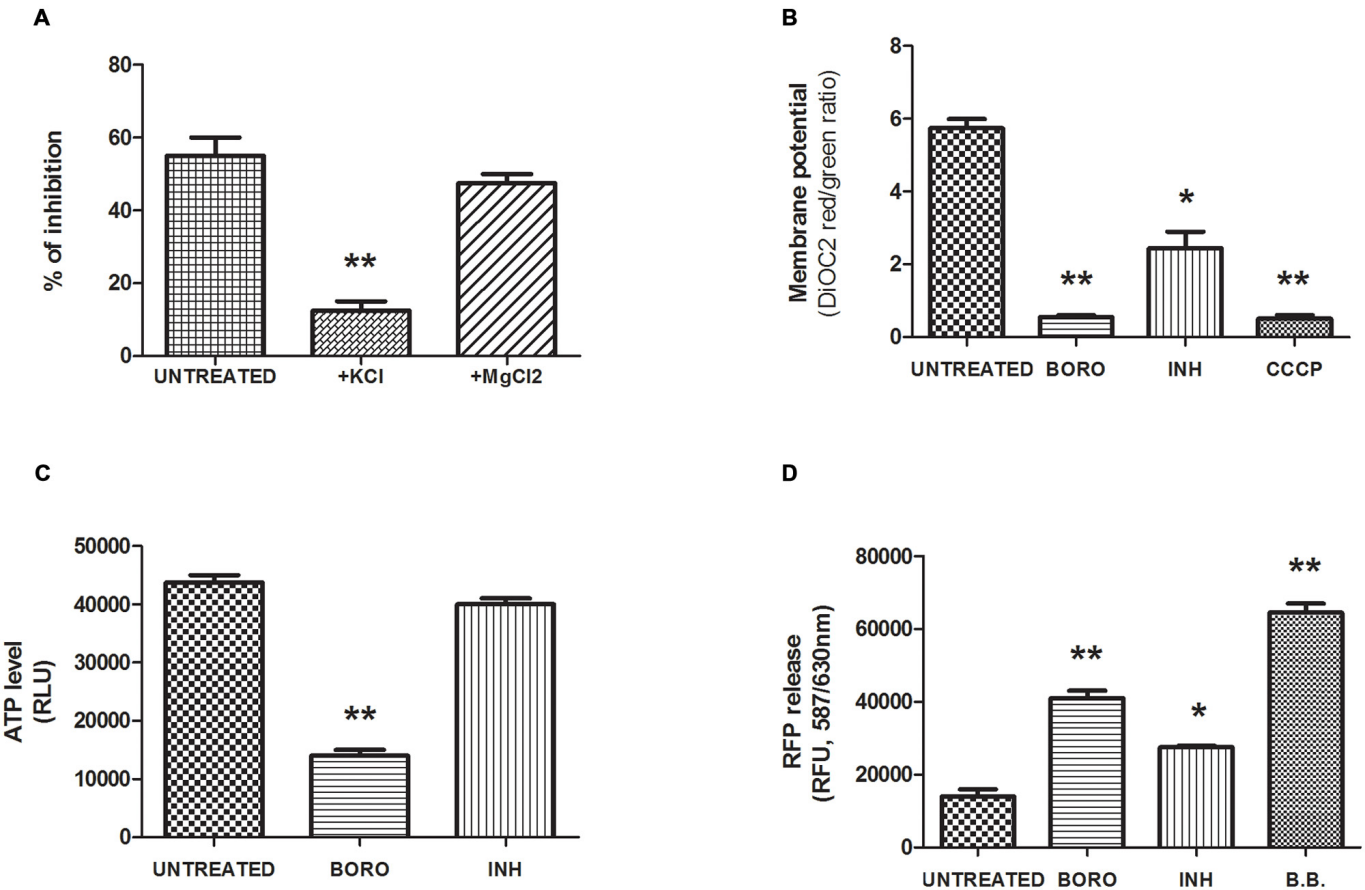
**Mechanism of action of boromycin. (A)** Effect of cations added to the medium on growth inhibition by boromycin. Exponentially growing *M. bovis* BCG culture was exposed to MIC_50_ (0.05 μM) boromycin either in standard medium (Mock) or to medium with added potassium chloride (+KCl, 20 mg/ml) or magnesium chloride (+MgCl_2_, 10 mg/ml). The effect on growth was determined by turbidity measurement after 5 days. Percent inhibition compared to boromycin-free control cultures grown in standard medium is shown. **(B)** Effect of boromycin on the membrane potential. Exponentially growing *M. bovis* BCG culture was exposed to MIC_90_ (0.2 μM) boromycin for 2 days after which the membrane potential was measured by the DiOC2, (3,3′-Diethyloxacarbocyanine Iodide) fluorescence assay (see Materials and Methods). Carbonyl cyanide m-chlorophenyl hydrazone (CCCP, 50 μM), positive control. INH, Isoniazid MIC_90_ (0.5 μM). **(C)** Effect of boromycin on the intracellular ATP level. Exponentially growing *M. bovis* BCG culture was exposed to MIC_90_ (0.2 μM) boromycin for 2 days after which the ATP content was measured was measured by ATP-dependent luciferin/luciferase assay (see Materials and Methods). INH, Isoniazid MIC_90_ (0.5 μM). RLU, relative luminescence unit. **(D)** Effect of boromycin on cytoplasmic protein leakage. Exponentially growing *M. bovis* BCG culture was exposed to MIC_90_ (0.2 μM) boromycin for 2 days after which release of Red fluorescence protein (RFP) was measured in the supernatant. INH, Isoniazid, MIC_90_ (0.5 μM). RFU, relative fluorescence unit. B.B: positive control, culture disrupted by bead beating. **(A–D)**: Shown are the averages of three independent experiments with standard deviations and Student *T*-test statistical significances given by a two-tailed P values with ^∗^*p* < 0.05 and ^∗∗^*p* < 0.01.

Boromycin was previously shown in *B. subtilis* to act as a potassium ionophore and to cause loss of the bacterium’s potassium ion gradient. Addition of potassium salt to the medium – but not addition of (divalent) magnesium salt blocked the growth inhibitor activity of the drug (Pache and Zahner, 1969). We tested the protective effect of high concentrations of potassium (20 mg/mL) or magnesium (10 mg/mL) cations in the culture media while subjecting *M. bovis* BCG to boromycin treatment at MIC_50_. In standard broth, we observed the expected 50% growth inhibition effect (**Figure 3A**). This growth inhibition was reduced to 10% in presence of KCl whereas it was unaffected by the addition of MgCl_2_ (**Figure 3A**). In contrast, addition of KCl did not affect growth inhibition by isoniazid (Supplementary Figure S2). These results suggest that boromycin’s mechanism of action against mycobacteria is similar to its actions against Gram positive bacteria, which is to say that the polyether macrolide appears to act as a potassium ionophore. We also tested whether the medium pH influenced the growth inhibitory activity of the antibiotic (pH 5.5, 6.4, and 9), which was not the case (Supplementary Figure S3), consistent with boromycin’s neutral (i.e., not involving protons) carrier property (Hutter et al., 1967).

If boromycin indeed acts as an ionophore and causes the collapse of the potassium gradient across the bacillus’ membrane we expect to see an effect of the compound on the membrane potential. To determine whether this is the case, we measured the effect of the antibiotic on the membrane potential employing the 3,3-Diethyloxacarbocyanine iodide (DiOC2) probe which accumulates in cells in a membrane potential-dependent manner (Novo et al., 2000). Upon accumulation, the dye forms aggregates that emit a red fluorescence signal (λ488/630 nm) whereas a reduced membrane potential prevents accumulation and results in green fluorescence emission (λ488/520 nm), that is the red/green ratio reflects the membrane potential status. **Figure 3B** shows that as expected, boromycin treatment caused a marked depolarization of the membrane as illustrated by the drop in the red/green ratio, similar to the effect of the uncoupler carbonyl cyanide m-chloro phenyl hydrazone (CCCP). In comparison, isoniazid treatment affected the membrane potential only moderately. To characterize the cellular consequences of boromycin exposure and collapse of the membrane potential further, we measured the impact of the ionophore on the cellular ATP level. ATP synthesis requires membrane polarization and we predicted that boromycin treatment would results in a marked reduction in ATP level. We used the BactiterGlo kit which makes use of the ATP-dependent luciferin/luciferase reaction to measure ATP. **Figure 3C** shows a drastic reduction of the ATP level upon boromycin exposure, which was not observed upon Isoniazid treatment as reported by others (Koul et al., 2014). Finally, we show in **Figure 3D** that boromycin treatment results in leakage of cytoplasmic protein. We employed a strain expressing a plasmid-encoded red fluorescent protein (λ587/630 nm) and subjected cultures to MIC_90_ of boromycin. We used centrifugation to pellet the treated cultures and measured the cell-content release, reflected by the red fluorescence signal measured in the supernatant. Cultures disrupted by bead beating were used as a positive control. Boromycin treatment correlated with a marked cell-content release as reflected by the increase in red fluorescence. Treatment with Isoniazid was associated with a moderate increase in the fluorescence signal. It has previously been suggested that isoniazid treatment leads to moderate cell lysis (Vilcheze et al., 2000).

## Discussion

Boromycin is a polyether macrolide antibiotic with selective activity against Gram positive bacteria where it acts as ionophore for potassium ions. The antibiotic is ineffective against Gram negative bacteria where the outer membrane appears to block access of the compound to the cytoplasmic membrane. Here we show boromycin is active against *Mycobacterium tuberculosis* which, similar to Gram negative bacteria, possesses an outer membrane. Boromycin is a potent, submicromolar inhibitor of mycobacterial growth with submicromolar bactericidal activity against growing and non-growing drug tolerant persister bacilli. Exposure to boromycin resulted in a loss of membrane potential, reduction of the intracellular ATP level and leakage of cytoplasmic protein. Consistent with boromycin acting as a potassium ionophore addition of KCl to the medium blocked its antimycobacterial activity. In contrast to the potent antimycobacterial activities cytotoxicity and haemolytic activity were low with a selectivity index of more than 300. Spontaneous resistant mutants could not be isolated indicating a very low spontaneous resistance mutation frequency of less than 10^-9^/CFU.

Ionophores are currently not being used in medicine mainly because of their generally poor selectivity. However, these antibiotics are widely in use in livestock veterinarian medicine (Russell and Strobel, 1989). It is interesting to note that this class of molecules recently gained attention as potential anti-cancer agents. Salinomycin, another polyether monovalent cation ionophore, has been shown to possess high specificity against cancer stem cells (Gupta et al., 2009) and has now entered clinical trial studies (Zhou et al., 2013). Similar compounds are actively considered for their antibacterial and anti-parasitic properties (Huczynski, 2012; Huczynski et al., 2012; Antoszczak et al., 2014a,b,c; D’Alessandro et al., 2015). Here we add to the growing list of potentially therapeutically interesting ionophores: we identified boromycin as a potent and selective antimycobacterial compound. Further studies will determine the efficacy of this new antimycobacterial in TB mouse models. Our findings also suggest that it may be worthwhile to consider a so far underexplored approach in TB drug discovery: targeting the mycobacterial cell membrane as opposed to specific macromolecules may represent an attractive strategy to slow down genetic resistance development. Importantly, this chemotherapeutic intervention level appears to be an effective way to kill drug tolerant persister bacilli. Therefore, membrane function targeting agents could contribute to the development of novel treatment shortening anti-TB combination regimens.

## Author Contributions

WM, DA, and TD designed the experiments, WM and DA carried out the experiments and WM and TD wrote the manuscript.

## Conflict of Interest Statement

The authors declare that the research was conducted in the absence of any commercial or financial relationships that could be construed as a potential conflict of interest.

## Abbreviations

BCG: Bacille Calmette–Guérin
BORO: boromycin
INH: Isoniazid
TB: tuberculosis
