# Supplementary material for: Boromycin Kills Mycobacterial Persisters without Detectable Resistance
Source: Front Microbiol. 2016 Feb 22;7:199. doi: 10.3389/fmicb.2016.00199 (PMC4761863; doi:10.3389/fmicb.2016.00199)
Supplement: Supplementary file 1 [file Presentation_1.PDF]

SUPPLEMENTARY FIGURES LEGENDS

SUPPLEMENTARY FIGURE 1: Boromycin bactericidal activity in Wayne's model of hypoxia. 20 days old M. bovis hypoxic cultures were exposed to MIC90 (0.2 µM) of boromycin and MIC90 of isoniazid (0.5 µM) for 5 days after which cfu were enumerated. Shown are the averages of two independent experiments with standard deviations. Student T-test statistical significances given by a two-tailed P values are displaed with *** p<0.001.

SUPPLEMENTARY FIGURE 2: Effect of cations added to the medium on isoniazid's growth inhibition activity. Exponentially growing M. bovis BCG culture was exposed to MIC50 (0.03 µM) boromycin either in standard medium (mock) or in medium with added potassium chloride (+KCl, 20 mg/ml) or magnesium chloride (+MgCl2, 10 mg/ml). The effect on growth was determined by turbidity measurement after 5 days. Percentage of inhibition compared to boromycin-free control cultures grown in standard medium is shown. Shown are the averages of three independent experiments with standard deviations.

SUPPLEMENTARY FIGURE 3: Effect of pH on growth boromycin's growth inhibition activity. Exponentially growing M. bovis BCG culture was exposed to MIC50 (0.03 µM) boromycin either in standard medium (Mock) or in medium with adjusted pH. The effect on growth was determined by turbidity measurement after 5 days. Percentage inhibition compared to boromycin-free control cultures grown in standard medium is shown. Shown are the averages of three independent experiments with standard deviations. Student T-test statistical significances given by a two-tailed P values with ** p<0.01

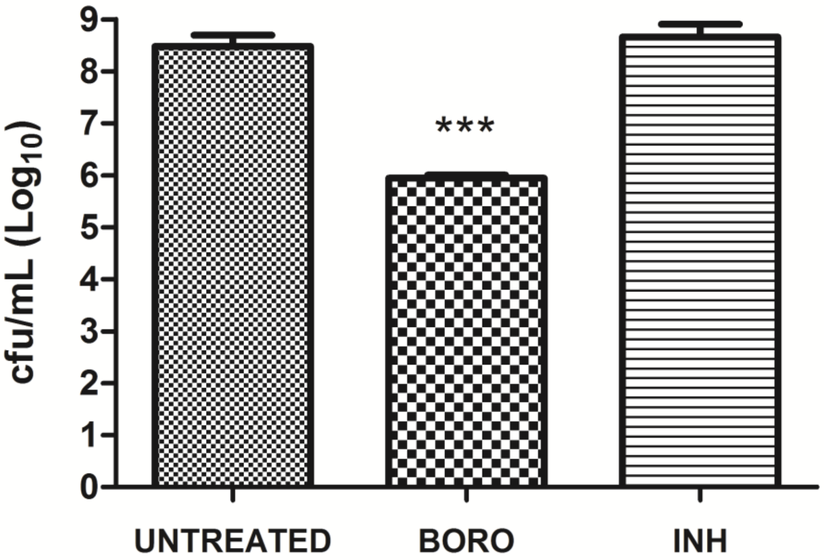

Supplementary Figure 1

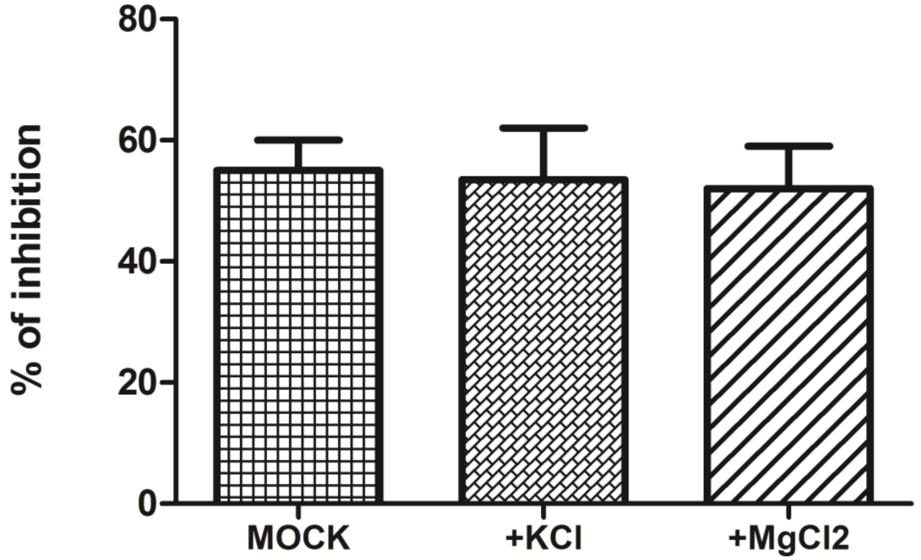

Supplementary Figure 2

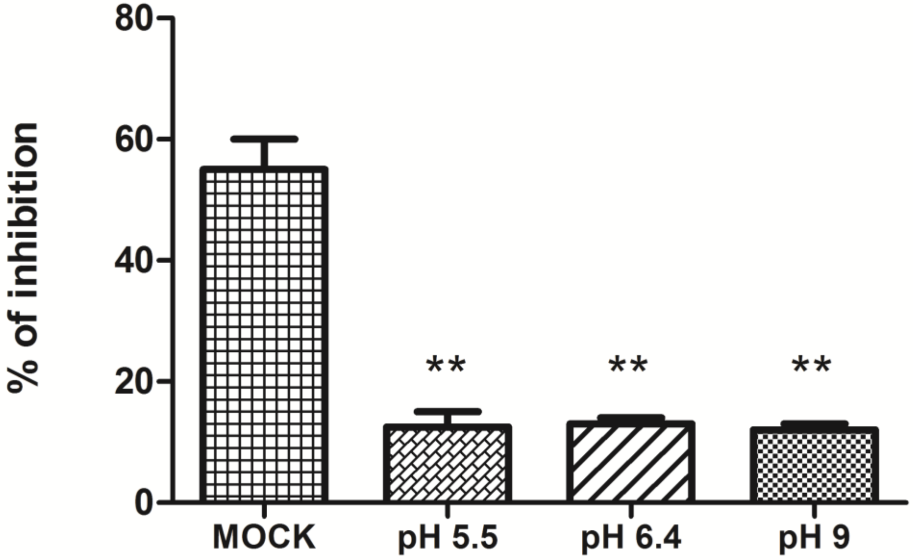

Supplementary Figure 3
